# Biological investigations of *Aspergillus ficuum* via in vivo*, *in vitro and in silico analyses

**DOI:** 10.1038/s41598-023-43819-y

**Published:** 2023-10-11

**Authors:** Zafar Ali Shah, Khalid Khan, Tanzeel Shah, Nasir Ahmad, Akhtar Muhammad, Haroon ur Rashid

**Affiliations:** 1 Department of Chemistry, Islamia College University, Peshawar, Khyber Pakhtunkhwa Pakistan; 2 Institute of Basic Medical Sciences, Khyber Medical University, Peshawar, Khyber Pakhtunkhwa Pakistan; 3 Center of Chemical, Pharmaceutical and Food Sciences, Federal University of Pelotas, Pelotas RS, Brazil; 4 Institute of Chemistry, Sao Paulo State University, Araraquara, Sao Paulo, Brazil

**Keywords:** Biochemistry, Biological techniques, Biotechnology, Computational biology and bioinformatics, Drug discovery

## Abstract

Serious human health impacts have been observed worldwide due to several life-threatening diseases such as cancer, candidiasis, hepatic coma, and gastritis etc. Exploration of nature for the treatment of such fatal diseases is an area of immense interest for the scientific community. Based on this idea, the genus *Aspergillus* was selected to discover its hidden therapeutic potential. The genus *Aspergillus* is known to possess several biologically active compounds. The current research aimed to assess the biological and pharmacological potency of the extracts of less-studied *Aspergillus ficuum* (FCBP-DNA-1266) (*A. ficuum*) employing experimental and bioinformatics approaches. The disc diffusion method was used for the antifungal investigation, and the MTT assay was performed to assess the anticancer effects. Mice were employed as an in vivo* model* to evaluate the antispasmodic effects. A standard spectrophotometric technique was applied to gauge the urease inhibitory activity. The antifungal studies indicate that both n-hexane and ethyl acetate extracts were significantly active against *Candida albicans* (*C. albicans*) with their zone of inhibitions (ZOI) values reported as 19 ± 1.06 mm and 25 ± 0.55 mm, respectively at a dose of 30 µg.mL^−1^. In vitro cytotoxicity assay against HeLa, fibroblast 3T3, prostate PC3, and breast MCF-7 cancer cell lines was performed. The ethyl acetate extract of *A. ficuum* was found to be significantly active against MCF-7 with its IC_50_ value of 43.88 µg.mL^−1^. However, no substantial effects on the percent cell death of HeLa cancer cell lines were observed. In addition, the *A. ficuum* extracts also inhibited the urease enzyme compared to standard thiourea. The antispasmodic activity of *A. ficuum* extract was assessed by an in vivo model and the results demonstrated promising activity at 150 mg.kg^−1^. Molecular docking results also supported the antifungal, anticancer, and antiurease potency of *A. ficuum* extract. Overall, the results display promising aspects of *A. ficuum* extract as a future pharmacological source.

## Introduction

New drugs are needed due to certain factors such as globalization, the aging population, and microbial resistance to existing medicines. Only a small number of biologically active compounds are currently used in prescription drugs^1^. Scientists are currently investigating emerging diseases to gain a better understanding of them and find their potential cures using various natural and chemical formulations. However, there are still many areas that remain unexplored due to insufficient knowledge and techniques. To find new bioactive compounds that can be used for medicinal, agricultural, and industrial purposes, researchers are focusing on isolating such compounds from new species of fungi^2^.

In recent times, researchers have isolated several metabolites from fungi, including anthraquinones, alkaloids, terpenes, steroids, flavonoids, and cyclic peptides. Some secondary metabolites produced by various fungi have been found to exhibit potent biological activities such as antifungal, antitumor, anti-inflammatory antibacterial, antiparasitic, and antiviral properties. The chemical diversity of fungal secondary metabolites provides a significant advantage for the development of new drugs^3,4^.

Anofinic acid, a potent active metabolite produced by *Aspergillus tubingensis*, has shown strong antimicrobial activity against a variety of hazardous bacteria in addition to its potent activity against various carcinoma cells, including Hep-G2, MCF-7, and HCT-116^2^. Sorbicillinoids possess the capability to be used as pharmaceutical and agrochemical agents due to their antiviral, antimicrobial, and anticancer effects besides their use as food colorants, and pigments. Sorbicillin and sorbicatechol D have been reported to exhibit antiproliferative effects against HT-29 tumor cells dose-dependently^5^. The metabolites gliotoxin, deoxytryptoquivaline, and patulin isolated from various *Aspergillus* species are known to induce apoptosis in cells^6–8^. A study performed on the secondary metabolites (terretonin N and butyrolactone I) of *A. terreus* confirmed their promising anticancer potential. Cellular apoptosis was induced at higher rates in cancer cells lacking a necrotic apoptotic pathway^9^. Similarly, recent studies on extracts of various fungi, particularly *Aspergillus,* have revealed their potent anticancer, antinociceptive, and anti-inflammatory activities^10,11^. In addition, the latest studies have also confirmed the strong enzyme-inhibiting effects of numerous species of fungi^9^.

*Aspergillus ficuum* is a filamentous fungus that belongs to the *Niger* clade in the *Aspergillus* section *Nigri*. Its morphological characteristics are the formation of spores and the display of colony characteristics. The taxonomy of the *Niger* clade depends on the generation of various secondary metabolites belonging to the five classes such as pyranone, alkaloid, cyclopentapeptide, polyketide, and sterol. These metabolites are known as naphthopyrones, malformins, bicoumarins (kotanins), fumonisin B2 and B4, citric acid, diketopiperazine, asperazine, and other related compounds^12^. Extensive biological aspects have been reported for the *Niger* clade^13^.

In our previous study, we investigated the metabolic profile of *A. ficuum* extracts for the first time, which led to the discovery of various bioactive compounds, including hydroxyvittatine, aurasperone D, choline sulfate, noruron, cetrimonium, heneicosane, kurilensoside, eicosane, and nonadecane. Furthermore, a pharmacological analysis of *A. ficuum* extracts was also carried out via in vitro and in vivo models^14^. In continuation of our previous study, extracts of the lesser-explored member of the Niger clade, *A. ficuum* were further investigated for their antifungal and anticancer activities via an in vitro model. Whereas their anti-inflammatory and antispasmodic effects were studied through an in vivo model. In addition, computational analyses were performed to support the in vitro and in vivo results. These investigations of *A. ficuum* extracts will offer new understandings into the area of the fungal diaspora.

## Materials and methods

### Chemicals and animals

All standard chemicals and solvents were obtained from Sigma Aldrich. The (BALB/c) mice weighing 25–35 g were purchased from the National Institute of Health (NIH), Islamabad, Pakistan. They were acclimated at 22 °C on a light/dark cycle (12 h/12 h) with adequate provision of water and food throughout the study. All methods were performed following the relevant guidelines and regulations^15^. The in vivo studies were conducted under the number 7196/LM/UoA Ethical Committee FAHV&S, the University of Agriculture Peshawar, Pakistan^14^.

### Fungal strain

The fungal strain (*A. ficuum*, FCBP-DNA-1266) was obtained from the fungal bank of the University of Punjab, Pakistan.

### Culture cultivation

The fungal culture was cultured at 28 °C for 21 days in the static state. *A. ficuum* spores (10^5^ conidia/mL) were transferred to the multiple Erlenmeyer flask (500 mL) containing about 250 mL of Potato Dextrose Broth (PDB). To inhibit bacterial growth in the medium, PDB was added with 25 mg.L^−1^ of streptomycin sulfate^16^.

### Extraction and fractionation

After 21 days, the mycelia formed in each flask were removed and processed for drying. The mycelia were washed several times with deionized water to remove any media ingredients. The constant dry weight of mycelia was obtained after drying at 60 °C in an oven. The dried mycelia were crushed to powder using a mortar and pestle. The powdered mycelia weighing 60 g was extracted in triplicate using three hundred milliliters of ethyl acetate (EtOAc) (3 × 300 mL). Subsequently, the obtained 4 g of ethyl acetate extract was fractionated by n-hexane (3 × 300 mL). Both portions were condensed via a rotary evaporator (Buchi, Germany, Model R-300). The dried-up portions, ethyl acetate (2.3 g) and n-hexane (1.7 g) were kept at 4 °C in the refrigerator for further studies^16,17^.

### Antifungal assay

The microorganisms such as *Aspergillus niger* (*A. niger*), *Fusarium oxysporum* (*F. oxysporum*), *Trichoderma harzianum* (*T. harzianum*)*, Candida albicans* (*C. albicans*)*,* and *Aspergillus flavus* (*A. flavus*) used for the antifungal assay were acquired from the Department of Agricultural Chemistry and Biochemistry, the University of Agriculture, Peshawar, Pakistan. A sterilized malt extract agar (MEA) medium was prepared and poured into Petri dishes. The spores from each fungal inoculum were added to these plates. The sterilized discs were soaked in *A. ficuum* extracts at concentrations of 10, 20, and 30 µg.mL^−1^ and were then positioned on the plates. Subsequently, the plates were cultured at 28 °C and then analyzed for antifungal assay after 72 h. Mancozeb was used as a drug control while DMSO was utilized as a negative control. The antifungal analysis was performed by measuring the zone of inhibition in millimeters^17^.

### Antispasmodic activity

Standard protocol was followed with minor modifications^18,19^. Mice weighing 20–30 g were used for the antispasmodic activity. Before the experiments, five mouse groups were created and kept on fast. Ethyl acetate extract of *A. ficuum* was introduced in triplicate at a dose of 10–20 mg.Kg^−1^. Two groups were orally administered castor oil (0.5 mL) and normal saline water (negative control) at a dose of 10 mL.kg^−1^. The residual three groups were orally administered *A. ficuum* extract at a dose of 10–20 mg.kg^−1^. Each group of mice was orally administered with charcoal (1 mL) after 1 h of induction of castor oil, and extract. The passage of charcoal from the pylorus to the caecum was noted by sacrificing mice through cervical dislocation after 50 min of charcoal induction. The percent inhibition caused by the *A. ficuum* extract was determined as under:

Percent inhibition = [Distance covered by charcoal/Total length of intestine] × 100.

### MTT assay

The anticancer studies were carried out at Hussain Ejaz Institute, Department of Chemistry, University of Karachi. A standard colorimetric MTT (3-[4, 5-dimethylthiazole-2-yl]-2, 5-diphenyl-tetrazolium bromide) analysis was performed to determine the cytotoxic potential of crude extract of *A. ficuum* by using 96-well flat-bottomed microplates^20^. In this study, HeLa cells (Cervical Cancer), 3T3 (mouse fibroblast), PC3 cells (Prostrate Cancer), and MCF-7 (Breast Cancer) cells were examined. These different cancer cells were cultivated in Minimum Essential Medium Eagle (MEME), added with fetal bovine serum (5%) (FBS), penicillin (100 IU.mL^−1^), and streptomycin (100 µg.mL^−1^) in flasks (75 cm^2^). The flasks were cultured at 37 °C in a CO_2_ (5%) incubator. The substantially growing cells were counted with a hemocytometer followed by dilution with a specific medium. A volume of 100 µL of each cell culture (HeLa, 3T3, MCF-7, and PC3) having 1 × 10^4^ cells/well was seeded into 96-well plates at a concentration of 100 µL/well. The cell cultures were incubated overnight and the medium was separated from them. A newly harvested medium (200 µL) was administered to the well of each cell culture along with different concentration doses of extract of *A. ficuum* (15–60 µg.mL^−1^). After incubation for 48 h, each well was added MTT solution (200 µL) having a concentration of 5 mg.mL^−1^. The cell cultures were further nurtured for 4 h. Later, DMSO (100 µL) was introduced to each well. The synthesis of formazan by the decrease of MTT was determined using a microplate reader (Spectra Max Plus, Molecular Devices, CA, USA) through absorbance maximum at a wavelength of 570 nm. Doxorubicin and DMSO were used as a positive and negative control, respectively. The 50% growth inhibition (IC_50_) was measured for cytotoxicity by using the straight-line equation y = mx + c. By using Soft-Max Pro software (Molecular Device, USA), the following formula was applied for the measurement of percent inhibition:

% inhibition = 100 − (mean of O.D of *A ficuum* extract − mean of O.D of negative control)/(mean of O.D of positive control – mean of O.D of negative control) *100).

### Urease inhibition assay

The urease inhibitory effect of the crude extracts of *A. ficuum* was evaluated by following the standard protocol with little modifications^21^. A solution of 20 µL of Jack bean urease (2.5 units/mL) and 50 µL of urea (100 mM) was prepared. The solution was dissolved in phosphate buffer, pH 8.2 [K_2_HPO_4_·3H_2_O (0.01 M), EDTA (1.0 m), and LiCl_2_ (0.01 M)] and was mixed with 30 µL of 250 µg.mL^−1^ concentrations of EtOAc and n-hexane extracts. The mixture was subjected to incubation at 37 °C in a 96-well plate for 10 min. The reaction mixture was incubated at 30 °C in a 96-well plate for 15 min. The indophenol method was used to determine the amount of ammonia production to measure the suppression of urease activity. The phenol reagent [phenol (1%), sodium nitroprusside (0.005%), 40 µL] and the alkaline reagent [60 µL, NaOH (0.5%), NaOCl (0.1%)] were added to each well. Thiourea (100 µg.mL^−1^) was taken as a standard and data were obtained in triplicate by measuring the absorption maximum at 630 nm. Percent inhibition was determined via the equation:

Percent inhibition = 100 − (OD *A. ficuum extracts*/OD control) × 100.

### In silico analysis

Mycocompounds (Fig. S1) tentatively reported for *A. ficuum* extracts^14^ were chosen for docking assay against heat shock protein 90 (hsp90) (PDB ID: 6CJI) of *C. albicans*, jack bean urease (PDB ID: 3LA4) and human HER2 protein (PDB ID: 3PP0) at a resolution of 1.64, 2.05 and 2.25 Å, respectively^17,22,23^. High-resolution X-ray crystal structures of the chosen proteins were obtained from the Protein Data Bank (http://www.rcsb.org/pdb). By using a preparation program integrated into the Molecular Operating Environment (MOE) software, protein molecules were optimized for docking study by eliminating water molecules, introducing missing hydrogen atoms, allocating the correct hybridization state to each atom in each residue, and fixing charges. Active sites of the designated proteins were located using the active site finder tool embedded in MOE software. Using the docking tool of the MOE software, mycocompounds were docked to the active binding sites of the chosen pathogenic proteins. For each molecule, 30 conformations were produced using specific torsion angles for all the rotatable bonds. The MOE-implanted London dock scoring function was used to determine the binding energy for each protein–ligand complex system^24^.

### Statistical analysis

Statistical analysis of all data obtained was performed using GraphPad Prism version 8.0. Results were evaluated using a mean n = 3 with standard deviation. Antifungal analyses were evaluated through one-way ANOVA while means were separated by applying LSD at *P* ≤ 0.05. Similarly, the results of the in vivo pharmacological investigation were evaluated through one-way ANOVA, followed by post hoc analysis (Dunnett’s test).

### Ethics approval

All experimental protocols were approved by the ethical committee FAHV&S, the University of Agriculture Peshawar, Pakistan under the number 7196/LM/UoA. All methods are reported following ARRIVE guidelines (https://arriveguidelines.org).

## Results and discussions

### Antifungal activity

The antifungal analysis of ethyl acetate and n-hexane extracts of *A. ficuum* was determined versus five fungal species (Table 1).

**Table 1:** Antifungal potential of ethyl acetate and n-hexane extracts of *Aspergillus ficuum.*

Fractions	Concentration µg.mL^−1^	*F. oxysporum*	*A. flavus*	*A. niger*	*T. harzianum*	*C. albicans*
Zone of Inhibition (mm)						
Ethyl acetate	10	18 ± 1.30^b^	15 ± 1.15^c^	12 ± 0.81^b^	16 ± 1.04^b^	18 ± 0.51^b^
	20	19 ± 1.22^b^	17 ± 1.12^b^	12 ± 0.85^b^	17 ± 1.00^b^	19 ± 0.55^b^
	30	22 ± 1.24^a^	20 ± 1.12^a^	15 ± 0.82^a^	19 ± 1.07^a^	25 ± 0.55^a^
n-Hexane	10	06 ± 1.54^c^	09 ± 0.51^e^	10 ± 1.38^b^	12 ± 1.62^c^	13 ± 1.11^c^
	20	06 ± 1.33^c^	09 ± 0.45^e^	12 ± 1.32^b^	12 ± 1.65^c^	14 ± 1.02^c^
	30	07 ± 1.23^c^	11 ± 0.47^d^	13 ± 1.32^b^	13 ± 1.72^c^	19 ± 1.06^b^
Drug control	30	28	26	26	28	28

The values are represented as mean n = 3 with standard deviation followed by letters (a, b, c) indicating a significant difference LSD at *P* ≤ 0.05.

**Figure 1:**
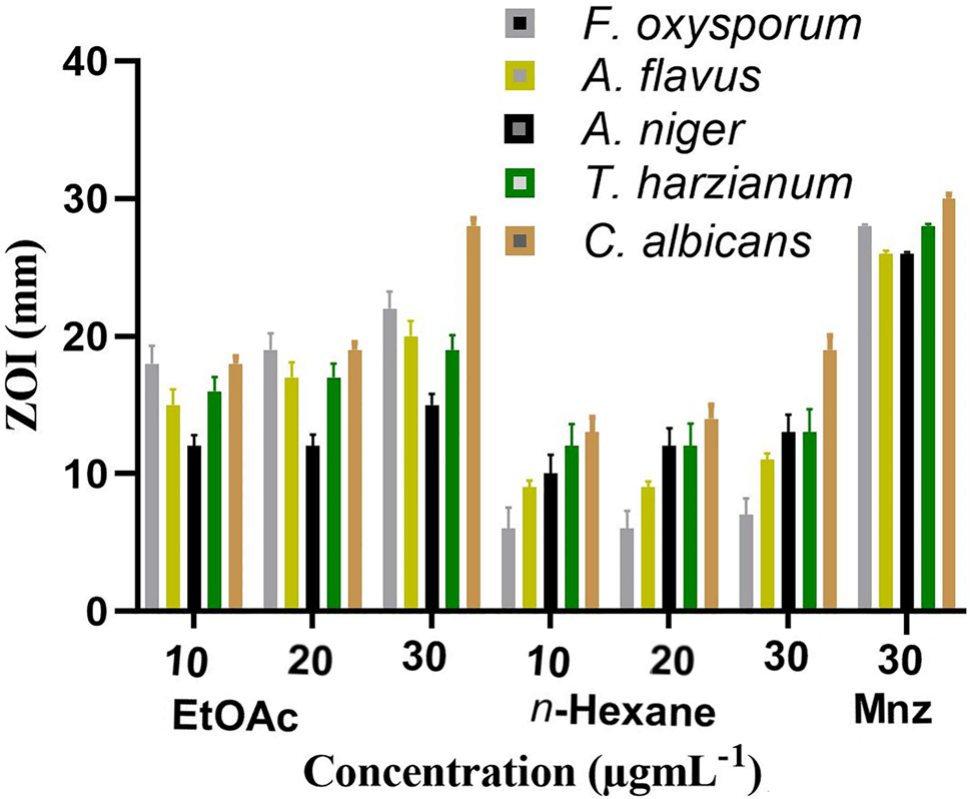
Graphical representation of antifungal activity.

*C. albicans* is a mutual fungus that causes death by colonizing the human skin and gastrointestinal tract. It is the causative agent of multidrug resistance in individuals with compromised immune systems due to multiple diseases. Several studies have been conducted on its control using natural products^25,26^. In this study, the antifungal investigations reveal that ethyl acetate extract was significantly active (*P* ≤ 0.05) against *C. albicans* with a ZOI of 25 ± 0.55, at a dose of 30 µg.mL^−1^. It was also recorded that ethyl acetate extract was substantially active (*P* ≤ 0.05) against all fungal species at a dose concentration of 30 µg.mL^−1^. Likewise, potent antifungal activity (*P* ≤ 0.01–0.05) was also exhibited by n-hexane extract against other fungal species (Fig. 1).

The n-hexane extract was also profoundly active (*P* ≤ 0.05) against the tested fungal strains at high concentrations. The fungal species are known to be great synthesizers of antifungal compounds. Several classes of compounds produced by the genus *Aspergillus*, such as statins and polyketides, are considered effective antifungal compounds^27^. A study conducted on the extract isolated from the endophytic fungus *Colletotrichum gloeosporioides* displayed potent antifungal activity against *C. albicans*^28^. In addition, a similar study was conducted on various fungal strains of different genera such as *Acremonium*, *Fusarium*, *Aspergillus,* and *Penicillium* which showed potent antifungal activities^29^. In another study, the n-hexane extract was found to be effective against *A. flavus* with a ZOI of 21.63 mm and less significant against *F. oxysporum* with a ZOI of 15.31 mm. The genus *Aspergillus* is considered a good producer of antimicrobial agents without any significant toxicity^30,31^.

### Antispasmodic activity

Inflammatory bowel disorders and gastritis are very common in humans and are induced by histamine and acetylcholine. The gastrointestinal tract becomes irritated and inflamed by smooth muscle contractions, causing uneasiness and discomfort. Such conditions are usually treated with antispasmodics which also show numerous side effects^18^. A charcoal motility assay was performed to assess the inhibition of ethyl acetate extract of *A. ficuum* (Table 2). The three different dosages of extracts from *A. ficuum* were able to show an antispasmodic effect. Smooth muscle contractions were reduced in a dose-dependent manner by ethyl acetate extract compared to mice treated with castor oil.

**Table 2:** The antispasmodic potential of ethyl acetate extract of *A. ficuum*.

Treatment	Dose	Total length of intestine (cm)	Distance covered by charcoal (cm)	Percent distance covered by charcoal
Control	10 (mg.kg^−1^)	47.2 ± 7.46	14.1 ± 1.16	29.87
Castor oil	10 (mL.kg^−1^)	48.4 ± 2.5	32.3 ± 1.41	66.73
Ethyl acetate extract of* A. ficuum*	10 (mg.kg^−1^)	44 ± 1	7.6 ± 1	17.27
	15 (mg.kg^−1^)	45 ± 0.57	10.2 ± 1	22.66
	20 (mg.kg^−1^)	45 ± 1	12.1 ± 1	26.88

**Figure 2:**
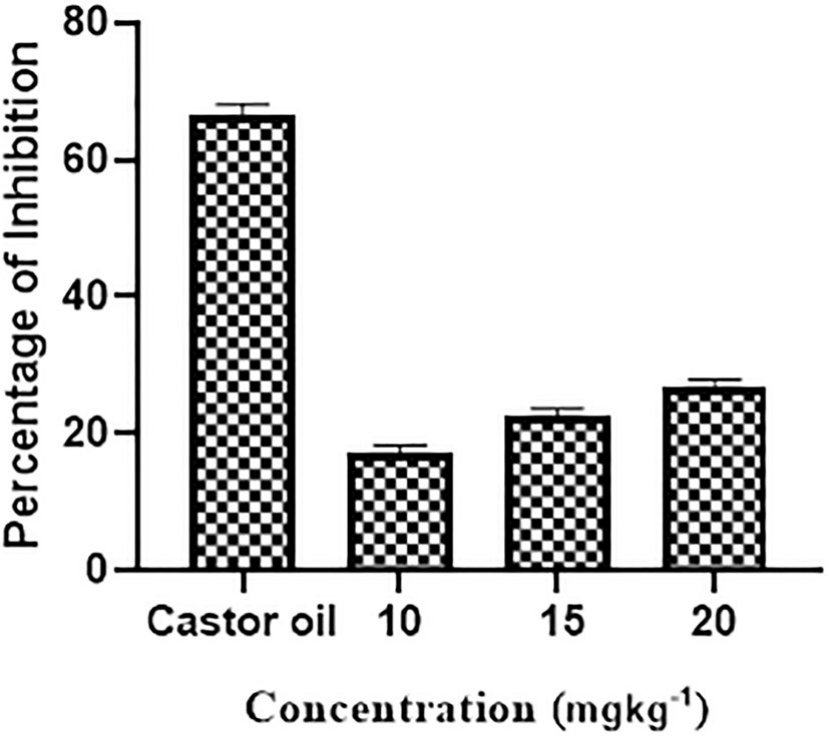
Antispasmodic activity of ethyl acetate extracts of *A. ficuum*.

It was observed that as concentrations of *A. ficuum* extract increased, there was a gradual increase in spasmodic activity, which is not a good indication. However, *A. ficuum* extracts possessed antispasmodic activity compared to the percent mobility of charcoal in castor oil, providing evidence that *A. ficuum* extracts can relieve spasms (Fig. 2). According to the literature, an antispasmodic effect against three different spasmogens was shown in a study of the n-hexane extract from endophytic fungi^32^. The literature review showed that bio-transformed diterpenes from *A. niger* displayed significant antispasmodic activity^33^. Although this study was conducted using a few concentrations of the extract, further investigation of the mycochemicals of this fungus will lead to the development of the most standardized and effective drug.

### Anticancer activity

The anticancer activity of ethyl acetate extracts of *A. ficuum* was measured versus HeLa, 3T3, PC3, and breast cancer cells (MCF-7) (Table 3).

**Table 3:** Different values of IC_50_ of *A. ficuum* extracts against various cancer cell lines.

Test sample	IC_50_ µg.mL^−1^			
	HeLa	3T3	PC3	MCF-7
*A. ficuum*	250.1	75.54	70.07	43.88

**Figure 3:**
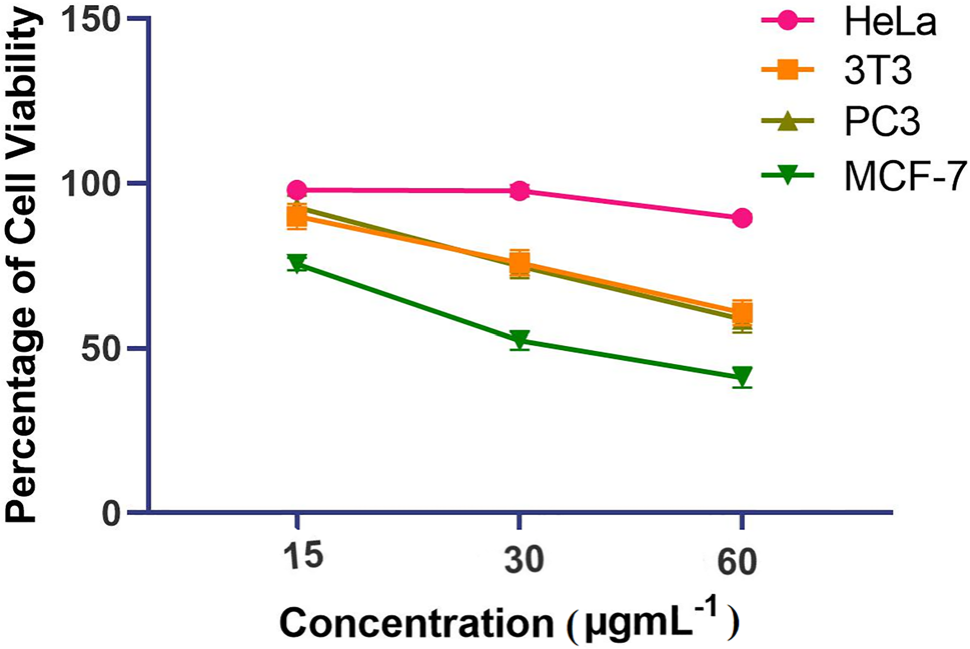
Percent cell viability of different cancer lines at different doses of ethyl acetate extract of *A. ficuum.*

In the case of MCF-7, the IC_50_ value was noted to be 43.88 µg.mL^−1^, significantly potent compared to other cancer cell lines, HeLa, 3T3 & PC3 with their IC_50_ noted to be 250.1, 75.54, and 70.07 µg.mL^−1^, respectively. From the analysis, it was visible that percent cell viability decreases with the increase in the concentration of the extract. The linear pattern of cell viability against the dose concentrations of the extracts was evident (Fig. 3).

**Figure 4:**
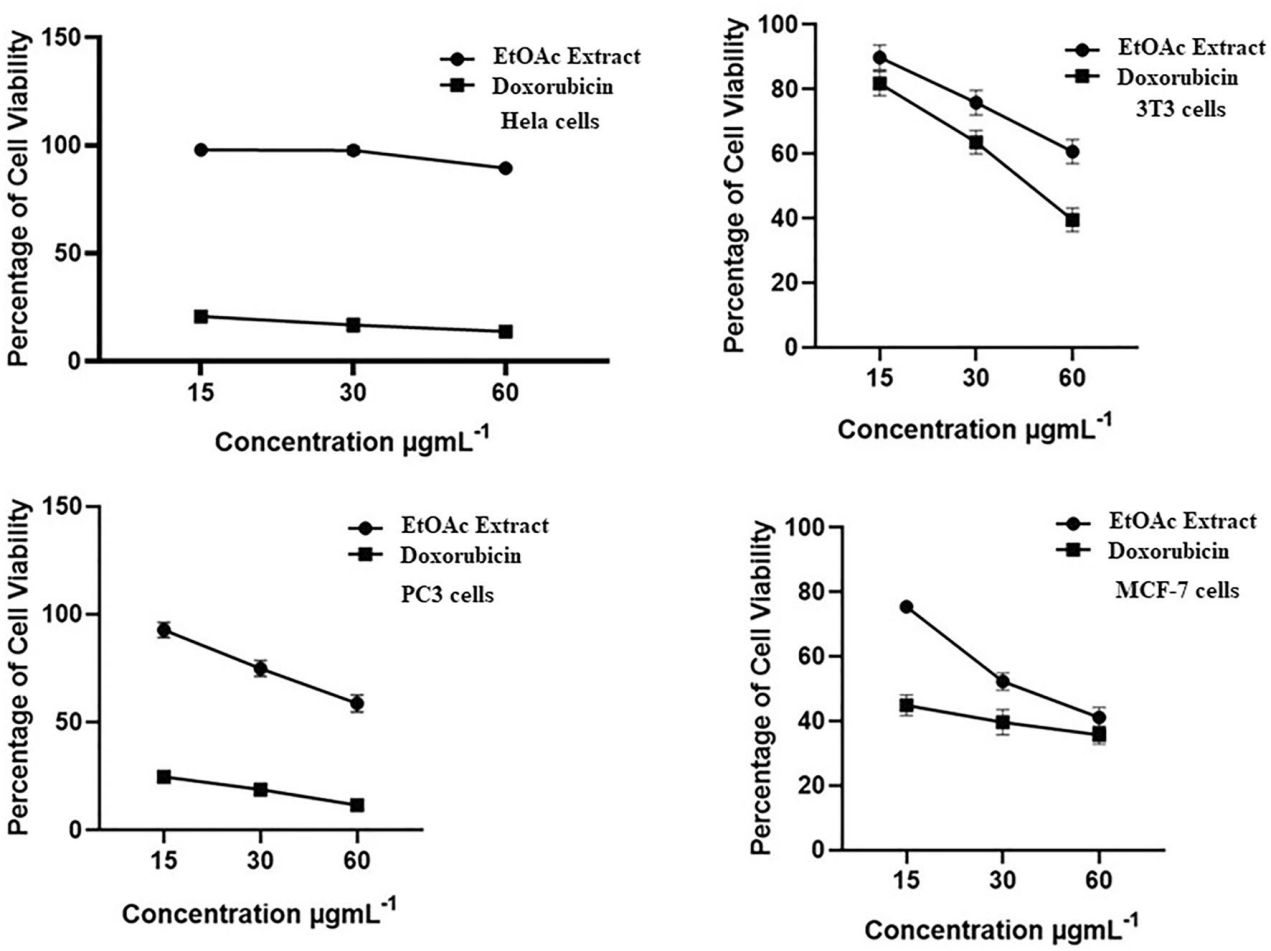
Comparative representation of percent cell viability of different cancer lines at several doses of ethyl acetate extract and standard drug Doxorubicin.

At a high dose of 60 µg.mL^−1^, no significant difference was observed for the positive control, doxorubicin. A similar pattern was observed for all doses of *A. ficuum* extract against the 3T3 cancer cell line. Moreover, no significant effect of *A. ficuum* extract was found against the HeLa cell line. It was also evident that high doses of *A. ficuum* extract caused significant cell death (Fig. 4).

Previously, a study showed that more than 100 anticancer drugs associated to 19 diverse chemical groups were obtained from fungi^34^. Artika et al*.* studied the anticancer activity of endophytic fungi. Their data showed that only one isolated chemical at a concentration of 400 µg.mL^−1^ exhibited potency comparable to the standard drug^35^. Felczykowska and coworkers found that fungi possess antiproliferative materials. They studied that the fungus, *Protoparmeliopsis muralis* isolated from lichen exhibited potent activity versus MCF-7 cancer cells^36^. Tincho et al*.* studied the cytotoxic potential of numerous fungi isolated from *Terminalia cattapa*. They discovered that an extract of *F. oxysporum* exhibited a potent 50 percent inhibitory potential versus cancer cell lines^37^. Thomas et al.examined twenty-one fungal extracts versus MCF-7 cancer cells. Their results revealed that sample F-21 possessed strong anticancer activity with its IC_50_ value of 44.75 µg.mL^−1^^38^. Nevertheless, several species of *Aspergillus* are highlighted as potent producers of anticancer metabolites^39^. The biosynthesis of secondary metabolites in fungi is highly reliant on the substrate and other ecological conditions^40^. Since this study was performed on a PDB medium, mycelial growth on other substrates and optimized conditions could contribute to the potent anticancer activities of fungal extracts.

### Urease inhibition assay

The conversion of urea into harmful products by the urease enzyme has serious effects on plants, animals, and humans. Its action causes urinary stones, gastritis, gastric cancer, hepatic coma, and other serious diseases in living organisms. The ureolytic activity of urease was suppressed by the natural products obtained from various sources^41^. The Urease inhibition assay of different extracts of *A. ficuum* was assessed in this study. A significant percent urease inhibitory potential was exhibited by ethyl acetate extract of *A. ficuum*. The ethyl acetate and n-hexane extracts of *A. ficuum* suppressed urease by 71.58 and 53.22%, respectively.

**Figure 5:**
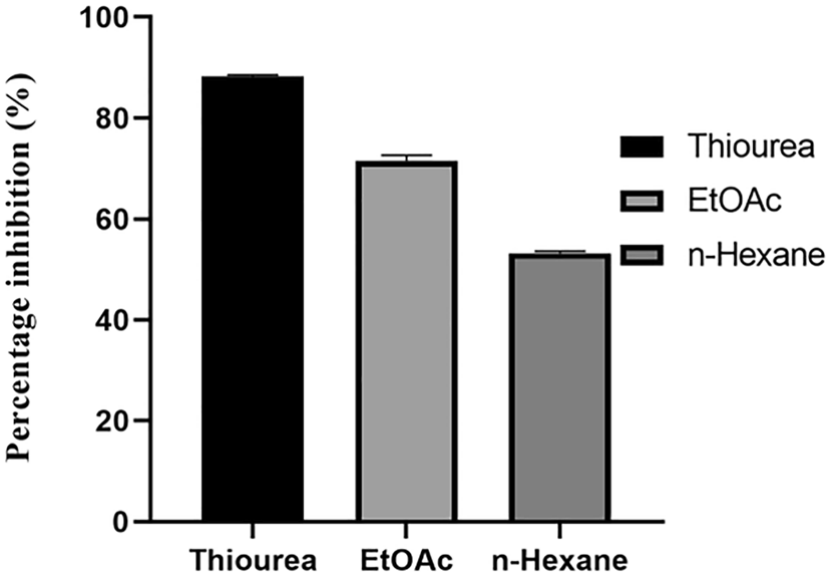
Urease inhibition assay of different extracts of *A. ficuum.*

The result agreed well with standard thiourea (Fig. 5). The difference between the ethyl acetate extract and the standard thiourea was not significant, which is a good sign as the commercially available ureases are toxic and less stable. The study regarding the suppression of the urease enzyme by fungi is very limited. Rauf et al.determined that the ethyl acetate fraction of *Screlotium rolfsii* and *A. flavus* was significantly inactive to urease enzymes with less than 50% inhibition^42^. A literature review suggested that fungal metabolites can inhibit the urease enzyme at different dose levels^43^. A research group isolated two compounds from *Paecilomyces formosus* which exhibited significant urease inhibitory activity with their potencies of 75.8 and 190.5 µg.mL^−1^^44^. Another group of researchers found the urease inhibitory activity of a metabolite isolated from fungi, Bipolaris sorokiniana LK12. In a dose-dependent study, the metabolites showed an IC_50_ value of 81.62 µg.mL^−1^ against the urease enzyme^45^.

### Docking analysis

**Figure 6:**
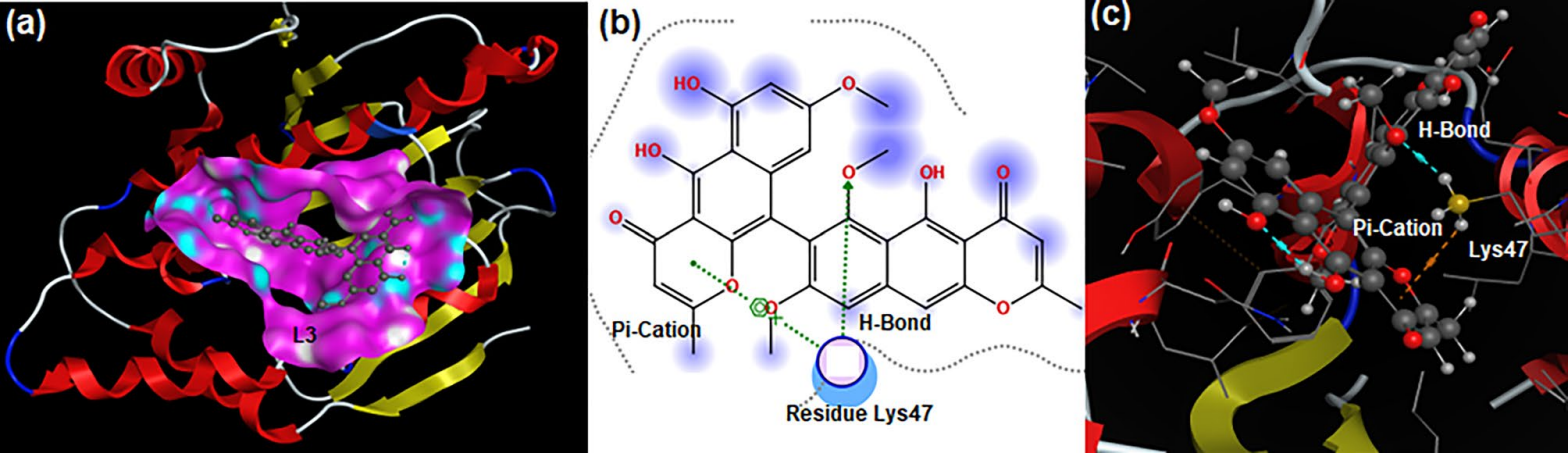
(**a**) 3D view of ligand **L3** in the active pocket of Hsp90 protein; (**b**) 2D and (**c**) 3D interactions of **L3**-Hsp90 protein complex.

Ethyl acetate extract of *A. ficuum* was found significantly active against all tested fungi in general and *C. albicans* in particular. Therefore, the mycocompounds were cautiously identified from ethyl acetate extract^14^ [Fig. S1] were docked versus the Hsp90 protein of *C. albicans* to support the antifungal activity of *A. ficuum* extract. Hsp90 plays a vital role in pathogenesis by performing protein biogenesis as well as interacting with various cellular proteins^46^ and is referred to as a molecular chaperone; its inhibition will halt fungal infection. Docking results reveal that all selected ligands (**L1-L9**) can inhibit the Hsp90 protein by forming a stable protein–ligand complex. Among all docked ligands, **L3** was found to be more efficient since it developed two physical interactions with the catalytic residues of Hsp90 resulting in the highest binding energy of − 7.9738 kcal.mol^−1^ (Table 4). One conventional H-bond was generated between residue Lys47 and the polar oxygen atom of **L3**; the second Pi-cation interaction was formed between the benzene ring of **L3** and residue Lys47 (Fig. 6). **L1** formed two physical interactions with Hsp90 causing a binding affinity of − 5.7161 kcal.mol^−1^ (Table 4). Catalytic residues Gly86 and Lys47 were involved in developing two conventional H-bonds with two different hydroxyl groups of **L1** (Fig. S2a,b). Ligand **L2** was associated with amino acid Asn95 of Hsp90 through one H-bond; it resulted in the binding energy of − 5.8924 kcal.mol^−1^ (Table 4) (Fig. S2c,d). **L4** formed a stable protein–ligand complex by developing two conventional H-bond interactions; two different oxygen atoms of the sulfonate group of **L4** were found associated with the catalytic residue Lys47 (Fig. S2e,f). The binding energy was noted to be − 4.8836 kcal.mol^−1^ (Table 4).

**Table 4:** Binding energy, number of interactions, nature of interactions, distances of interactions, and interacting residues of Hsp90 protein (6CJI) of *C. albicans* against the selected ligands.

Ligands	Binding energy (kcal.mol^−1^)	Number of interactions	Nature of interactions	Distances of interactions	Interacting residues
L1	− 5.7161	02	H-Bond	3.09	Gly86
			H-Bond	2.90	Lys47
L2	− 5.8924	01	H-Bond	2.96	Asn95
L3	− 7.9738	03	H-Bond	3.04	Lys47
			Pi-Cation	3.33	Lys47
L4	− 4.8836	02	H-Bond	3.07	Lys47
			H-Bond	3.06	Lys47
L5	− 7.8284	02	H-Bond	3.03	Gly86
			H-Bond	3.09	Lys47
L6	− 6.7134	02	H-Bond	2.75	Asp43
			H-Bond	3.33	Asp43
L7	− 7.5397	03	H-Bond	3.01	Glu36
			H-Bond	2.99	Gly126
			H-Bond	3.14	Lys47
L8	− 7.7921	02	H-Bond	2.80	Asp91
			H-Bond	3.19	Lys47
L9	− 7.0975	02	H-Bond	3.37	Asp91
			H-Bond	3.77	Asp91

**L5** bound to the active sites of Hsp90 generated the second-highest binding affinity of − 7.8284 kcal.mol^−1^ (Table 4) among all screened ligands. Residue Lys47 developed an H-bond interaction with the carbonyl oxygen atom of **L5**; the second H-bond originated between residue Lys47 and the NH group of **L5** (Fig. S2g,h). **L6** was linked with Hsp90 through two H-bond interactions originating binding affinity of − 6.7134 kcal.mol^−1^ (Table 4). Residue Asp43 formed two H-bonds with two different hydroxyl groups of **L6** (Fig. S2i,j). Ligand **L7** formed a stable protein–ligand complex through three H-bond interactions. The Hydroxyl moiety of **L7** participated in developing H-bond interaction with residue Glu36. Two different carbonyl groups of **L7** formed two H-bond interactions with residue Lys47 and Gly126, respectively (Fig. S2k,l). The binding energy value was calculated to be − 7.5397 kcal.mol^−1^ (Table 4). **L8** formed two physical interactions with Hsp90 originating with the third highest binding affinity of -7.7921 kcal.mol^−1^ (Table 4). One H-bond was developed between residue Asp91 and the hydroxyl moiety of **L8**; a second H-bond was generated between the carbonyl oxygen atom of **L8** and residue Lys47 (Fig. S2m, n). In the case of **L9**, two physical forces were responsible for the formation of a protein–ligand complex; the binding energy value was noted to be − 7.0975 kcal.mol^−1^ (Table 4). Catalytic residue Asp91 generated two conventional H-bond interactions with **L9** (Fig. S2o,p). Docking data display three major findings: firstly, all ligands (**L1-L9**) can bind to catalytic residues located in the active pocket of Hsp90; secondly, **L3** has the highest binding affinity with Hsp90 among all screened ligands; thirdly, residue Lys47 is actively involved in binding to six ligands. Keeping in view the chemistry of Lys47, future drug candidates can be designed. These findings support the antifungal activity of *A. ficuum* extracts*.*

**Figure 7:**
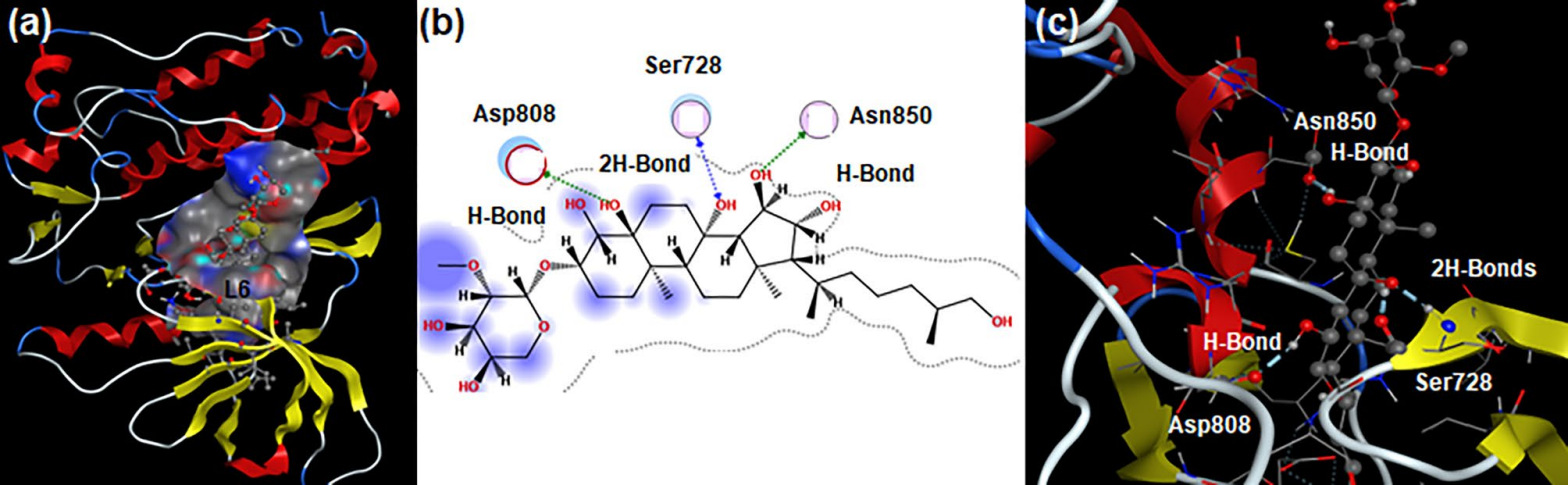
(**a**) 3D view of ligand **L6** in the active pocket of Her-2 protein; (**b**) 2D and (**c**) 3D interactions of the **L6**-HER2 protein complex.

Similarly, ligands (**L1-L9**) were docked against the HER2 protein to support the anticancer activity of *A. ficuum* extracts*.* HER2 is a membrane tyrosine kinase enzyme; it plays a major part in tumor growth and the spreading of breast cancer on activation^47^. Docking results indicate that ligand **L6** showed the highest affinity toward the HER2 protein among all screened ligands. It is associated with HER2 protein through four H-Bond interactions resulting in the highest binding energy of − 7.8907 kcal.mol^−1^ (Table 5). Residue Ser728 developed two H-Bond interactions with the same hydroxyl group of **L6**; each amino acid Asn850 and Asp808 formed a single H-Bond interaction with two different hydroxyl groups of **L6** (Fig. 7). Ligand **L1** generated three physical interactions with HER2 protein stemming binding affinity of − 5.2498 kcal.mol^−1^ (Table 5). Catalytic residue Asp808 participated in developing two H-Bond interactions with the same hydrogen atom of the hydroxyl moiety of **L1**. Residue Ser728 formed Pi-H interaction with the benzene ring of **L1** (Fig. S3a,b). **L2** developed two non-covalent interactions with HER2 protein; these interactions originated a binding affinity of − 5.1722 kcal.mol^−1^ (Table 5). Each of the residues (Cys805 and Ser728) formed a single H-bond with the polar hydrogen atom of the NH moiety and the carbonyl oxygen atom of **L2**, respectively (Fig. S3c,d).

**Table 5:** Binding energy, number of interactions, nature of interactions, distances of interactions, and interacting residues of HER2 protein (3PP0) of humans against the selected ligands.

Ligands	Binding energy (kcal.mol^−1^)	Number of interactions	Nature of interactions	Distances of interactions	Interacting residues
L1	− 5.2498	03	H-Bond	2.97	Asp808
			H-Bond	3.14	Asp808
			Pi-H	4.11	Ser728
L2	− 5.1722	02	H-Bond	3.97	Cys805
			H-Bond	3.38	Ser728
L3	− 6.8174	02	H-Bond	2.97	Leu726
			H-Bond	3.03	Cys805
L4	− 6.1479	01	H-Bond	3.22	Thr862
L5	− 7.7000	02	H-Bond	3.10	Ser728
			Pi-H	4.02	Gly727
L6	− 7.8907	04	H-Bond	2.71	Asp808
			H-Bond	2.67	Ser728
			H-Bond	2.72	Asn850
			H-Bond	2.78	Ser728
L7	− 6.5073	03	H-Bond	2.70	Ser728
			H-Bond	3.10	Ser728
			H-Bond	2.90	Lys724
L8	− 6.8651	01	H-Bond	3.11	Asp808
L9	− 6.7778	02	H-Bond	3.39	Asp808
			Pi-Cation	3.75	Asp808

Ligand **L3** formed a stable protein–ligand complex by developing two physical interactions with binding affinity reported to be − 6.8174 kcal.mol^−1^ (Table 5). Each of the amino acids (Leu726 and Cys805) generated a single H-Bond with the polar hydrogen of the hydroxyl moiety and carbonyl oxygen atom of **L3**, respectively (Fig. S3e,f). **L4** generated a single interaction with the HER2 protein resulting in a binding affinity of − 6.1479 kcal.mol^−1^ (Table 5). Residue Thr862 formed a single H-Bond interaction with the polar hydrogen of the CH_2_ group of **L4** (Fig. S3g,h). Ligand **L5** was associated with HER2 protein through two non-covalent interactions; these forces generated the second-highest binding energy of − 7.7000 kcal.mol^−1^ (Table 5). Catalytic residue Ser728 formed a single H-Bond interaction with the oxygen atom of the methoxy group of **L5**; residue Gly727 developed a single Pi-H interaction with the pyrrole moiety of **L5** (Fig. S3i,j). Ligand **L7** formed a stable protein–ligand complex by generating two physical interactions; it resulted in a binding energy of − 6.5073 kcal.mol^−1^ (Table 5). Residue Ser728 originated two H-Bond interactions with the polar hydrogen atom of the hydroxyl group and carbonyl oxygen atom of **L7**; Lys724 developed a single H-Bond with the hydroxyl moiety of **L7** (Fig. S3k,l).

**L8** displayed single H-Bond interaction with HER2 protein producing the third highest binding affinity of − 6.8651 kcal.mol^−1^ (Table 5). The Hydroxyl moiety of **L8** was involved in the creation of the protein–ligand complex (Fig. S3m,n). Ligand **L9** generated two physical interactions with HER2 protein; these non-covalent forces resulted in a binding energy value of − 6.7778 kcal.mol^−1^ (Table 5). Residue Asp808 developed H-Bond and Pi-Cation interactions with two different methyl groups of **L9**, respectively (Fig. S3o,p)^48^. In the case of HER2 protein, three new aspects emerged: firstly, all ligands (**L1-L9**) form a stable protein–ligand complex; secondly, ligand **L6** has a greater ability to bind to the active pocket of HER2 protein among all tested ligands; thirdly, the catalytic role of residue Ser728 is found prominently by binding to five ligands. Structural features of **L6** and residue Ser728 should be considered for drugs designed against breast cancer. Herein, docking results support the anticancer activity of *A. ficuum* extracts.

**Figure 8:**
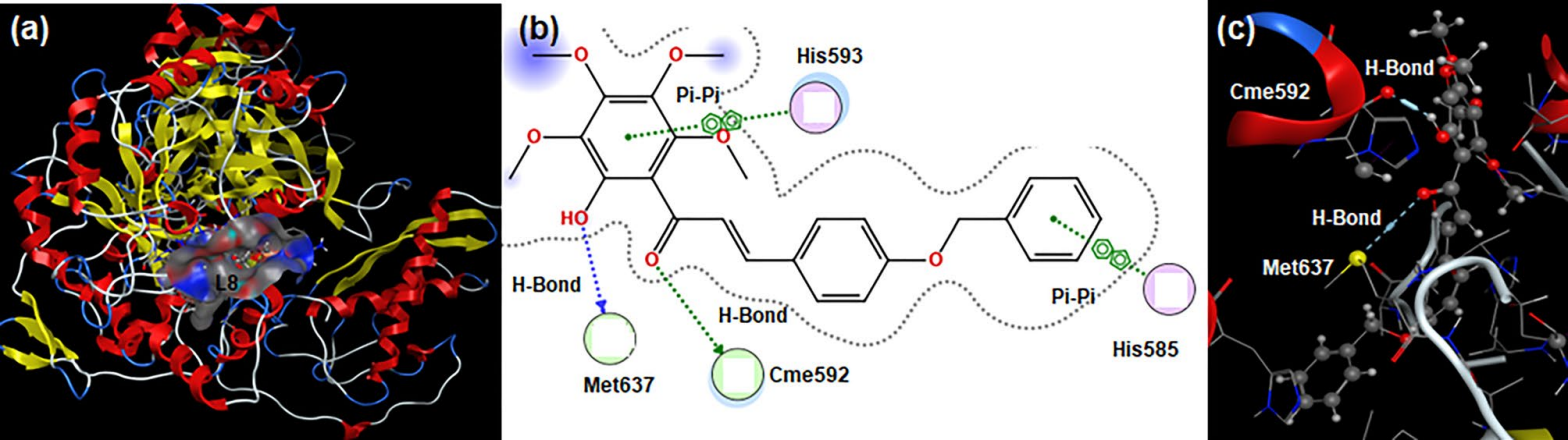
(**a**) 3D view of ligand **L8** in the active pocket of urease protein; (**b**) 2D and (**c**) 3D interactions of **L6**-Urease protein complex.

Similarly, ligands (**L1-L9**) were also screened against the urease enzyme through the molecular docking technique; the results indicated that all ligands could inhibit the urease enzyme by forming a stable protein–ligand complex. Based on binding affinity, **L8** was found to have the highest binding affinity of − 7.7921 kcal.mol^−1^ (Table 6) among all selected ligands. It developed four physical interactions with urease protein. Both residues (His593 and His585) generated a single Pi–Pi interaction with two different benzene rings of **L8**; in addition, two residues namely Met637 and Cme592 also formed a single H-Bond interaction with the hydroxyl moiety and carbonyl oxygen atom of **L8**, respectively (Fig. 8). **L1** originated two non-covalent interactions with the urease enzyme resulting in the binding energy of − 5.0588 kcal.mol^−1^ (Table 6). Residue Arg835 developed a single H-Bond interaction with the hydroxyl moiety of **L1**; Arg575 formed a single H-Bond interaction with the methyl moiety of the heterocyclic ring of **L1** (Fig. S4a,b). Ligand **L2** generated a single non-covalent interaction with the urease enzyme; it resulted in a binding energy value of − 5.4100 kcal.mol^−1^ (Table 6). Catalytic residue Val831 participated in the development of a single H-Bond interaction with the carbonyl oxygen of **L2** (Fig. S4c,d).

**Table 6:** Binding energy, number of interactions, nature of interactions, distances of interactions, and interacting residues of urease protein (3LA4) of jack bean against the selected ligands.

Ligands	Binding energy (kcal.mol^−1^)	Number of interactions	Nature of interactions	Distances of interactions	Interacting residues
L1	− 5.0588	02	H-Bond	3.44	Arg575
			H-Bond	3.08	Arg835
L2	− 5.4100	01	H-Bond	3.26	Val831
L3	− 6.9148	02	Pi-H	3.48	Arg835
			Pi-Cation	4.00	Arg835
L4	− 4.5903	03	H-Bond	3.41	Glu584
			H-Bond	3.38	Glu584
			H-Bond	3.16	Gln649
L5	− 7.6472	02	H-Bond	3.43	Glu642
			H-Bond	3.41	Val831
L6	− 7.1083	01	H-Bond	3.32	Arg835
L7	− 7.5593	03	H-Bond	2.91	Ser645
			H-Bond	3.24	Thr829
			Pi-H	3.66	Arg835
L8	− 7.7921	04	H-Bond	3.91	Met637
			H-Bond	2.71	Cme592
			Pi-Pi	3.48	His585
			Pi-Pi	3.15	His593
L9	− 6.7125	02	H-Bond	3.29	Glu642
			H-Bond	3.35	Glu642

**L3** formed a stable protein–ligand complex by developing two physical interactions resulting in the binding affinity of − 6.9148 kcal.mol^−1^ (Table 6). Residue Arg835 generated Pi-H and Pi-Cation interactions with the benzene ring and oxygen heterocyclic ring of **L3,** respectively (Fig. S4e,f). **L4** generated four physical forces with the urease enzyme; these forces resulted in a binding affinity of − 4.5903 kcal.mol^−1^ (Table 6). Residue Glu584 developed two H-Bond interactions with two different methyl groups of **L4**. Gln649 formed a single H-Bond interaction with the oxygen atom of **L4** (Fig. S4g,h). Ligand **L5** was involved in complex formation with urease through two physical interactions; these interactions originated the second-highest binding affinity of − 7.6472 kcal.mol^−1^ (Table 6). Both residues (Glu642 and Val831) developed single H-Bond interactions with OH and NH groups of L5, respectively (Fig. S4i,j).

Ligand **L6** developed a single non-covalent interaction with the active spot of the urease enzyme yielding a binding affinity of − 7.1083 kcal.mol^−1^ (Table 6). Residue Arg835 displayed a single H-Bond interaction with the hydroxyl moiety of **L6** (Fig. S4k,l). **L7** showed three physical interactions with catalytic residues of urease enzyme generating the third highest binding energy of − 7.5593 kcal.mol^−1^ (Table 6). Two residues, namely, Ser645 and Thr829 formed single H-bond interaction with two different hydroxyl groups of **L7**; catalytic residue Arg835 displayed Pi-H interaction with the benzene ring of **L7** (Fig. S4m,n). Ligand **L9** was noticed attached to the active site of urease through two physical interactions that resulted in a binding affinity value of − 6.7125 kcal.mol^−1^ (Table 6). Catalytic residue Glu642 showed two H-Bond interactions with two different methyl groups of **L9** (Fig. S4o,p). Some interesting findings were obtained from docking data: all ligands form a stable protein–ligand complex with urease enzyme; **L8** shows the highest binding affinity among all screened ligands and catalytic residue Arg835 is noticed to be more active by forming a protein–ligand complex with four ligands.

## Conclusion

In this research, urease inhibition, antifungal, anticancer, and antispasmodic activities of *A. ficuum* extracts were explored for the first time by applying in vivo and in vitro models followed by in silico models. Like other fungi, both n-hexane and ethyl acetate extracts of *A. ficuum* were noticed active against five tested fungal strains. However, both extracts displayed significant activity against the *C. albicans.* Potent antispasmodic, as well as urease inhibitory activities, were recorded for *A. ficuum* extracts*.* Similarly, the promising anticancer effect of *A. ficuum* against PC3, 3T3, and especially MCF-7 cancer cell lines was documented. Furthermore, the antiurease, antifungal, and anticancer activities of *A. ficuum* extracts were supported by molecular docking results. Among all docked ligands, **L3, L6,** and **L8** displayed the highest binding affinity for Hsp90, HER2, and urease proteins, respectively. Similarly residues namely, Lys47, Ser728, and Arg835 in the active pockets of Hsp90, HER2, and urease proteins, respectively emerged as catalytically active residues generating H-Bond interactions with a greater number of ligands. Keeping in view the structural features of ligands (**L3, L6,** and **L8**) and residues (Lys47, Ser728, and Arg835), potential drug candidates could be designed in the future for the inhibition of pathogenic proteins such as Hsp90, HER2, and urease. In addition, the cultivation of *A. ficuum* extracts in a controlled environment with the progress of new approaches will lead to the discovery of new bioactive chemical scaffolds in the drug discovery process.

### Supplementary Information

Supplementary Information.

## Data Availability

All data generated or analyzed during this study are included in this published article and its supplementary information files.
