# Supplementary material for: Biological investigations of Aspergillus ficuum via in vivo, in vitro and in silico analyses
Source: Sci Rep. 2023 Oct 11;13:17260. doi: 10.1038/s41598-023-43819-y (PMC10570320; doi:10.1038/s41598-023-43819-y)
Supplement: Supplementary file 1 — Supplementary Information. [file 41598_2023_43819_MOESM1_ESM.docx]

**Supplementary Materials**

**Biological Investigations of *Aspergillus ficuum* via *In vivo, In vitro* and *In silico* Analyses**

**Zafar Ali Shah^1,2^, Khalid Khan^1*^, Tanzeel Shah^2^, Nasir Ahmad^1^, Akhtar Muhammad^1^, Haroon ur Rashid^3*^**

^1^**^*^**Department of Chemistry, Islamia College, Peshawar, KP, Pakistan

^2^Institute of Basic Medical Sciences, Khyber Medical University, Peshawar, KP, Pakistan

^3^ Department of Chemistry, Sao Paulo State University, Araraquara, Sao Paulo, Brazil

**^*^Corresponding authors:** drkhalidchem@yahoo.com (K.Khan), haroongold@gmail.com (H.ur.Rashid)

**Fig. S1.** Structures of tentatively identified ligands (L1-L9).


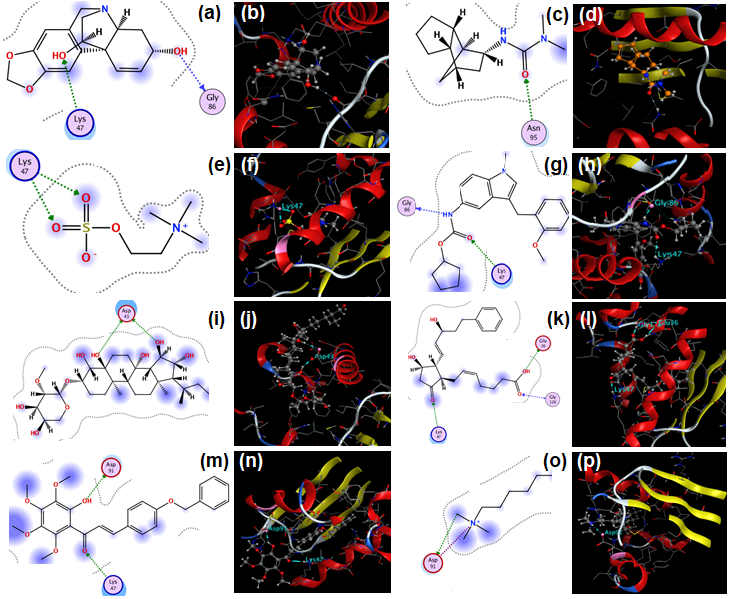


**Fig. S2.** (a) 2D and (b) 3D interactions of L1-Hsp90 complex; (c) 2D and (d) 3D interactions of L2-Hsp90 complex; (e) 2D and (f) 3D interactions of L4-Hsp90 complex; (g) 2D and (h) 3D interactions of L5-Hsp90 complex; (i) 2D and (j) 3D interactions of L6-Hsp90 complex; (k) 2D and (l) 3D interactions of L7-Hsp90 complex; (m) 2D and (n) 3D interactions of L8-Hsp90 complex; (o) 2D and (p) 3D interactions of L9-Hsp90 complex.


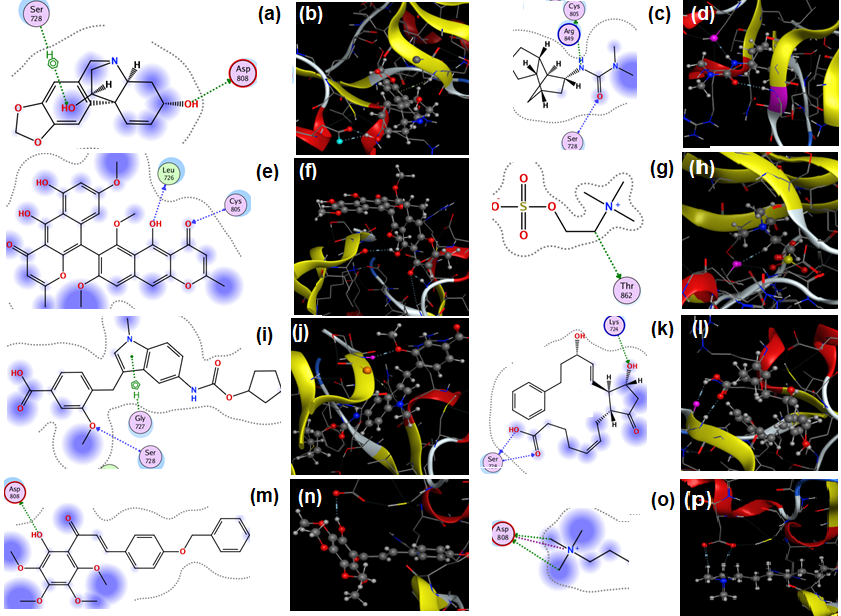


**Fig. S3.** (a) 2D and (b) 3D interactions of L1- HER2 complex; (c) 2D and (d) 3D interactions of L2-HER2 complex; (e) 2D and (f) 3D interactions of L3-HER2 complex; (g) 2D and (h) 3D interactions of L4-HER2 complex; (i) 2D and (j) 3D interactions of L5-HER2 complex; (k) 2D and (l) 3D interactions of L7-HER2 complex; (m) 2D and (n) 3D interactions of L8-HER2 complex; (o) 2D and (p) 3D interactions of L9-HER2 complex.


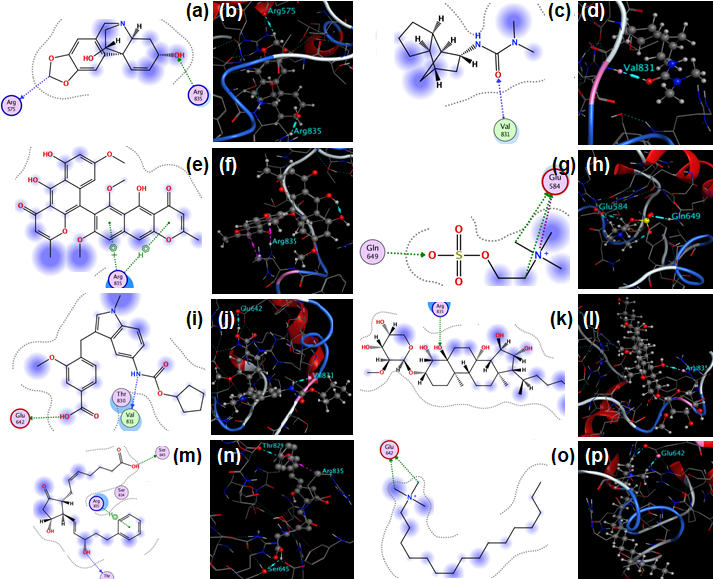


**Fig. S4.** (a) 2D and (b) 3D interactions of L1-Urease complex; (c) 2D and (d) 3D interactions of L2-Urease complex; (e) 2D and (f) 3D interactions of L3-Urease complex; (g) 2D and (h) 3D interactions of L4-Urease complex; (i) 2D and (j) 3D interactions of L5-Urease complex; (k) 2D and (l) 3D interactions of L6-Urease complex; (m) 2D and (n) 3D interactions of L7-Urease complex; (o) 2D and (p) 3D interactions of L9-Urease complex.
